# A Holistic Response to Musculoskeletal Health: Implications for Global Health Policy: Comment on "From Local Action to Global Policy: A Comparative Policy Content Analysis of National Policies to Address Musculoskeletal Health to Inform Global Policy Development"

**DOI:** 10.34172/ijhpm.8798

**Published:** 2025-04-08

**Authors:** Dorothy Lall

**Affiliations:** Community Health Department, Christian Medical College, Vellore, India.

**Keywords:** Musculoskeletal Health, Policy, Primary Care

## Abstract

This commentary is in response to the paper titled "A Comparative Policy Content Analysis of National Policies to Address Musculoskeletal Health to Inform Global Policy Development" by Schneider et al. This well-done policy content analysis identifies key themes and transferable principles to guide policy development for musculoskeletal health. In this commentary, I argue that the findings of this policy analysis should be used to develop global policies within the current framing of non-communicable diseases (NCDs), especially considering the growing burden of multimorbidity. The second point concerns the application of the building blocks framework and limitations in the use of the framework. Lastly, in this paper, I miss the needed emphasis for a global health policy that specifies primary healthcare and universal health coverage for a life course approach and an equitable response.

## Introduction

 The paper by Schneider and colleagues draws attention and focus to available policies for improving musculoskeletal health globally.^1^ In this paper, the authors identify and review policies from different countries to guide global policy development for musculoskeletal health. They conduct a detailed policy content analysis to draw major themes and identify transferable principles that could guide policy-makers to develop international and country-specific policies to address musculoskeletal health.

## Call to Action on Musculoskeletal Health

 The paper rightly calls for action towards developing policies to improve musculoskeletal health as a growing concern worldwide. The 2015 report on ageing and health identifies musculoskeletal conditions as a global threat to healthy ageing.^2^ As the authors point out, there is an increasing body of literature regarding the burden of disease even in low- and middle-income countries; however, few policies were identified, and none were from low- and middle-income countries. The methods used to determine policy documents were robust and thorough, using the Arksey and Mallory framework as well as reaching out to the Global Alliance for Musculoskeletal Health International Coordinating Council members and policy researchers (expert round), an e Delphi method and the use of snowballing to include several experts in the field across the globe.^3^ The authors point to the relative lack of attention to musculoskeletal health issues as a non-communicable disease (NCD).

## Musculoskeletal health, Non-communicable Disease and Multimorbidity

 It is interesting to note that all the policies included in the review, addressed musculoskeletal health issues outside the ambit of NCDs. The agenda for the control of NCDs has received much attention, and an international framing that has been propelled in part by the high-level meetings of the United Nations and inclusion as a sustainable development goal.^4^ However, musculoskeletal health issues have historically not been included in the conditions listed as priority or major NCDs. There has been much debate about framing NCDs and their usefulness, especially in the context of multimorbidity and person-centered care. There is a good argument for a more holistic frame that considers the ecosocial theory and approaches based on the social sciences that may provide an integrated way forward.^5^ A similar review of policies that address an integrated approach for NCDs among Member States of the Organization for Economic Co-operation and Development found that all the measures included in NCD policies are relevant to musculoskeletal health issues.^6,7^ The isolated focus of a global health policy for musculoskeletal conditions as a separate group of conditions may be counterproductive to a holistic, integrated response. I would argue that there is a case for inclusion of musculoskeletal conditions in policies that address NCDs in addition to a global policy for musculoskeletal health conditions. The danger of a policy that is not situated in the larger problem of NCDs is that it may lead to a verticalized health system response that dissuades holistic integrated care.

 In support of this argument, I would like to highlight the growing problem of multimorbidity. Multimorbidity has been defined as the existence of two or more conditions, most often chronic NCDs, in an individual.^8^ Multimorbidity is a challenge for health systems worldwide, which tend to address health issues in isolation and are not geared to managing multiple conditions in the same individual.^9^ Studies report that Musculoskeletal health problems are a recurring component of multimorbid conditions and frequently occur with diabetes, hypertension and other major NCDs.^10-12^ Therefore, policies for musculoskeletal conditions should also respond to NCDs and vice a versa policies for NCDs should include musculoskeletal health conditions given its growing burden as a co morbidity.

## Framework to Develop Policies for an Integrated Response to Musculoskeletal Health

 The authors use the building blocks framework to analyze and abstract themes and actionable principles recommended for musculoskeletal health policy development. The building blocks framework was found to be a good fit, and the authors justify its use after conducting a round of inductive coding of 6 policy documents as well as due to the familiarity of policy-makers with the framework. However, I would like to draw attention to the nature of recommendations resulting from the use of this framework and the lack of an exploration of the interconnections in the policy analysis. The use of this high-level framework has rendered the sub-themes and the transferable principles generic rather than nuanced for musculoskeletal health in the global health context. A close examination of the sub-themes and the principles highlights that these are all relevant to overall strengthening of the health system, irrespective of the health issue being addressed. Equity, person-centred care, complete packages from prevention to rehabilitation, multidisciplinary care, and geographic access to care listed in the service delivery category could apply to all health issues. These principles have been highlighted in multiple frameworks for health system strengthening, including the World Health Organization (WHO)-integrated people-centered health services framework.^13^ Further, the building blocks framework is useful when the interconnections between the building blocks and the implications of the interconnectedness of the building blocks are also considered. In their article on strengthening global health systems for a response to COVID-19, Borghi and Brown also use the building blocks framework to give directions for global health action. The authors highlight the relative lack of weighting the building blocks, for example governance is clearly a critical block underpinning all other building blocks.^14^. Borghi and Brown also highlight the need for complexity science in to complement the use of the building blocks framework to shape global action. In the way the framework has been applied by Schneider et al, there is no discussion of how these principles relate to each other and its implications for the global health system. This may have been a valuable addition of the analysis and discussion of the review. Another drawback in the application of the framework as siloed blocks is the considerable overlap in some of the principles that have been abstracted for example, in service delivery and workforce.

 Lastly, while the authors point out the lack of a life course approach, conspicuous by its absence in the selected policy documents, the discussion is also missing the needed emphasis on primary care as a policy direction for global action to improve access to services for musculoskeletal conditions. Primary healthcare and Universal health coverage are important elements of global health policy relevant to a life course approach and an equitable response to musculoskeletal health in the context of multimorbidity.^15,16^

 In summary, while this paper is an excellent resource for policy-makers and draws attention to the need for countries to develop policies for musculoskeletal health, the inclusion of musculoskeletal health in the NCD agenda, an integrated approach to multimorbidity, and a policy level emphasis on strengthening primary care are essential elements that should also be considered in the drafting of policy action for musculoskeletal health at both national and global levels.

## Ethical issues

 Not applicable.

## Conflicts of interest

 Author declares that she has no conflicts of interest.
